# Morphometric, haematological and physio-biochemical characterization of Bactrian (*Camelus bactrianus*) camel at high altitude

**DOI:** 10.1186/s12917-020-02481-6

**Published:** 2020-08-14

**Authors:** Dolker Lamo, Geeta Gahlawat, Sunil Kumar, Vijay K. Bharti, Puneet Ranjan, Deepak Kumar, Om Prakash Chaurasia

**Affiliations:** DRDO-Defence Institute of High Altitude Research (DIHAR), Leh, Ladakh, UT-194101 India

**Keywords:** Bactrian camel, Biochemical, Haematological, High altitude, Morphometric, Physiological

## Abstract

**Background:**

Biochemical and haematological parameters have not been determined in Bactrian camels kept at high altitude. Therefore, this study was undertaken to characterise different physiological, haematological, biochemical, and morphometric parameters of Bactrian camels of high altitude. For this, total fourteen high altitude healthy Bactrian camels were selected from Leh-Ladakh, India, a high altitude area, and thereafter divided into three age groups (*N* = 3 young; *N* = 6 adult; *N* = 5 old camels) to characterise for above parameters. All the results were compared with Lowlander Bactrian camels.

**Results:**

Morphometric measurement showed significant difference in body height, body length, front-hump height and girth, back-hump height and girth, abdomen girth, neck length, and circumference of the shank in the young age group camels as compared to other age groups of Bactrian camels (*p* < 0.05). Furthermore, all the physiological and haematological parameters were similar in all the age groups of camels (*p* < 0.05). However, the leukocyte, erythrocyte, Hb, platelets, monocyte, and ESR level were towards the higher side of the normal reference range of Lowlander Bactrian camels. Whereas, the biochemical analysis revealed a significant increase in triglycerides and decrease in protein levels in the younger age group as compared to other age groups (*p* < 0.05). Although, albumin, aspartate aminotransferase, iron, magnesium, urea, and creatinine levels were insignificant among the different groups, but observed towards the higher side of the low altitude reference range. Interestingly, the glucose levels in all the groups were observed towards the lower side of the range, which showed metabolic adaptation to high altitude.

**Conclusion:**

These findings suggested there is morphometric and biochemical variation in Bactrian camel of high altitude. The results further helped in establishing novel reference ranges for these parameters in Highlander Bactrian camel. Hence, this study will be the basis of future research on a Bactrian camel from high-altitude cold desert and helpful for better camel husbandry and health management in high altitude.

## Research highlights

➢ First report on morphometric, haematological and physio-biochemical profiling of Bactrian camel at high altitude

➢ Bactrian camel has haematological and biochemical adaptation to high altitude conditions

➢ Increase in erythrocytes and Hb to carry more oxygen for normal metabolic function

➢ Increase in segmented and banded neutrophils count to counter high altitude stress

➢ Hypoglycemia is an indication of increased dependency on non-glucose energy substrates

## Background

There are three species of camels in the world, i.e. single hump (*Camelus dromedarius*), double hump (*Camelus bactrianus*), and wild Bactrian camel (*Camelus ferus*). Single hump camels or Dromedary is found in many countries in low altitude areas, whereas double hump camels or Bactrian are generally found in Central Asia, Western China, and India in high altitude regions [1, 2]. In India, Bactrian camels are found in the high-altitude cold desert region of Nubra Valley, Partapur, Leh-Ladakh where temperature ranges from + 30 °C to − 25 °C [3]. The Highlander wild Bactrian camels were the ancestors of the domesticated ones which were bred for a long period generation-to-generation, and therefore, adapted to the high altitude climate. Thus Bactrian camels can withstand hypoxic conditions, severe cold, and extreme heat up to 30 °C prevalent in high-altitude cold desert of Ladakh.

The high altitude region is characterised by low oxygen pressure, extreme temperature variation of − 35 °C to 40 °C, low humidity, and high UV-radiation. Our earlier findings indicated hypobaric hypoxia is the most important stress factor at high altitude, which causes physiological stress on different species of domestic animals’ viz. goat, cattle, poultry, and ponies, and alters their metabolic functions [4–7]. Hence, it is considered that hypobaric hypoxia would also adversely affect blood biochemical and haematological parameters, an important indicator of the animals’ health status. Therefore, it is hypothesised that double hump camels of high altitude may have different physiological, haematological, and biochemical profile as compared to low altitude double hump camel reference ranges. Although, few researchers have reported the effect of sex, age, and lactation on haematological and biochemical parameters in Bactrian camel of the plain regions like Turkey and Iran [1, 8–10]. However, no studies have so far been done on camel physiology pertaining to Physio-biochemical parameters of Bactrian camels of high-altitude cold desert.

Furthermore, few reports indicated that Bactrian camels have some unique adaptability to these stress conditions as depicted by their good reproductive behaviour, load carrying capacity, exercise endurance, and disease resistance, despite poor nutrition at high altitude [1, 8, 11]. Further, these camels are known for their ability to endure long periods of travel without water carrying weight up to 200 to 250 kgs on the hilly rough terrain. However, other pack animals such as mules and ponies can carry only limited quantities of around 70 Kg load [7]. Hence, local farmers are more dependent on Bactrian camels in high-altitude areas for load carrying, dairy products, and other logistics. These camels have also gained significant attention by Indian army personnel of their use as pack or saddle animals at high altitude. However, populations of these camels are not increasing because the farmer has difficulty in husbandry and health management. As, there is no information on health monitoring and breed characterisation data, and so, veterinary clinicians and farmers face difficulties in scientific rearing and health management using the reference range of a single hump camel. Therefore, suitable breed conservation programme and commercial camel farming cannot be established despite having importance in load carrying and medicinal value of their milk and dairy products. Hence, animal husbandry and health management specific to these camels are difficult, thus further causing one of the important factors leads this camel into an endangered category.

Therefore, analysis of blood haematology, biochemistry, and morphometric measurement can help the veterinary clinician for accurate evaluation of the growth, health status, and selection of elite animals for civil and military use [12]. Thus, there is a need to characterise physiological responses, morphometric measurements, haematological, and biochemical parameters in double-humped camels from high-altitude cold desert. Eventually, this study will be further helpful in establishing a novel baseline of preliminary reference values for biochemical and haematological parameters of Highlander Bactrian camels.

## Results

There was no significant variation in respiration rate, heart rate, skin, and rectal temperature in the different age groups of high altitude Bactrian camels (*p* > 0.05; Table 1). The heart rate showed a tendency towards higher side in young age group, but the difference was not statistically significant as compared to other age groups of high altitude camel (*p* > 0.05). Moreover, all the high altitude groups have a higher respiration rate as compared to reference range of Lowlander camels (Table 1). The body and rectal temperature of the high altitude groups varied from 28.2 to 31.0 °C and 32.6 to 35.2 °C, respectively (Table 1). There was a significant difference in body height, body length, height of front-hump, girth of front-hump, height of back-hump, girth of back-hump, girth of abdomen, neck length and circumference of the shank in the young age group as compared to other two age groups of Bactrian camels (*p* < 0.05; Table 2). However, the face length, distance between the eyes, and the thickness of abdomen and shank skin were comparable in all the age groups, although no significant difference was observed in these parameters.

**Table 1:** Mean (±SEM) and reference ranges of physiological parameters in Bactrian camels at high altitude

S. No.	Physiological Parameters	Young age (2–4 years)	Adult age (5–10 years)	Old age (11–20 years)	Reference range at low altitude^a^	Reference range at high altitude^b^
1	Heart Rate (beats/min)	37.6 ± 0.88	35 ± 0.44	34 ± 1.67	30.0–45.0	30.0–40.0
2	Respiration Rate (beats/min)	12.6 ± 1.33	11.8 ± 0.16	11.6 ± 0.97	5.0–12.0	8.0–14.0
3	Body Temperature (°C)	29.6 ± 1.2	31 ± 0.68	28.2 ± 1.11	NR	24.0–32.0
4	Rectal Temperature (°C)	32.6 ± 0.66	34.8 ± 0.40	35.2 ± 1.31	32.0–35.0	32.0–38.0

^a^Reference ranges taken from Elitok and Cirak, (2018) ^b^ Reference range calculated based on present study; *NR* Not Reported; *SEM* Standard Error Mean

**Table 2:** Mean (±SEM) and reference ranges of morphological parameters in Bactrian camels at high altitude

S. No.	Morphological Parameters	Young age (2–4 years)	Adult age (5–10 years)	Old age (11–20 years)	Reference range at high altitude^**#**^
1	Height at withers (inches)	64.5 ± 0.28^a^	65.16 ± 0.64^ab^	67.4 ± 0.51^b^	63.0–69.0
2	Body Length (inches)	56.6 ± 2.33^a^	62.5 ± 1.60^ab^	65.2 ± 1.95^b^	52.0–71.0
3	Height of front hump (inches)	10.37 ± 1.44^a^	15.08 ± 0.61^b^	17.3 ± 1.31^b^	8.0–20.0
4	Girth of front hump (inches)	30 ± 1.0^a^	37.9 ± 1.5^b^	34.9 ± 1.32^ab^	29.0–42.0
5	Thickness of abdomen skin (cm)	1.03 ± ±0.16	1.00 ± 0.06	0.9176 ± 0.12	0.56–1.29
6	Girth of abdomen (inches)	76.3 ± 3.28^a^	83.66 ± 1.91^ab^	87.2 ± 3.12^b^	70.0–96.0
7	Circumference of shank (inches)	31.5 ± 0.5^a^	37 ± 1.18^b^	37.8 ± 1.01^b^	30.5–42.0
8	Thickness of shank skin (cm)	1.05 ± 0.18	1.03 ± 0.13	1.21 ± 0.11	0.49–1.53
9	Neck length (inches)	33.6 ± 0.88^a^	38.08 ± 1.0^b^	38.2 ± 1.01^b^	32.0–42.0
10	Face length (inches)	15.5 ± 0.5	16.16 ± 0.83	18.9 ± 0.92	14.0–21.0
11	Distance b/w eyes (inches)	10.26 ± 0.14	11 ± 0.57	10.9 ± 0.51	9.0–12.0
12	Back hump height (inches)	9.66 ± 1.33^a^	14.5 ± 0.5^b^	15.6 ± 1.29^b^	7.0–20.50
13	Back hump girth (inches)	30.33 ± 0.88^a^	45.41 ± 0.77^b^	43.8 ± 2.2^b^	29.0–50.0

# Reference range calculated from present study, as no literature are available on morphological parameters; SEM- Standard Error Mean; ^a,b^ - Mean values bearing the same superscripts in a column do not differ significantly (*p* > 0.05)

**Fig. 1:**
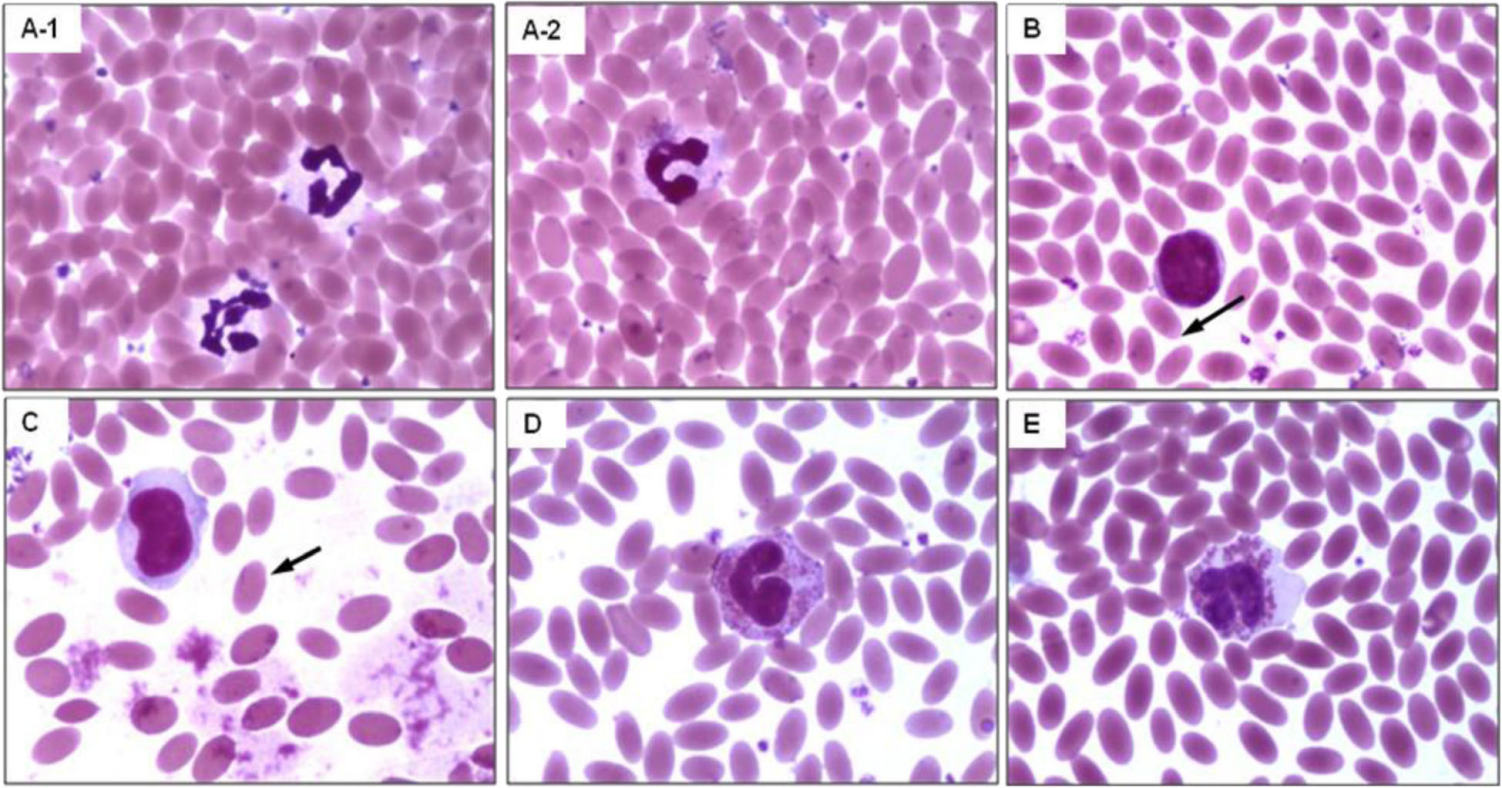
Morphology of different types of leukocyte, **a-**1) Segmented neutrophils; **a-**2 and **a-**3) Banded shaped neutrophils; **b-**1) Mature lymphocytes; **b-**2) Immature lymphocytes; **c**) Monocytes; **d**) Eosinophils; **e-**1 and **e-**2) Basophils; along with oval and elliptical shaped erythrocyte (arrow sign) in adult age groups of Bactrian camels (Wright-Giemsa; Original magnification 1000 x)

The haematological parameters viz. Total erythrocyte count, haemoglobin, packed cell volume (PCV), erythrocyte sedimentation rate (ESR), total leukocyte and platelets showed no significant changes among the different age groups (*p* < 0.05; Table 3). However, leukocyte level, platelet count, monocyte percentage, PCV, and ESR in all the age groups were observed towards the higher side of the reference range of high altitude groups as compared to the Lowlander Bactrian camel range (Table 3). Total erythrocyte counts in high altitude Bactrian camels were observed between 9.9 × 10^6^ to 11.6 × 10^6^ cells/mm^3^, whereas, their size was 6.39 × 3.41 μm with an area of 17.05 μm^2^ (Fig. 1). Further, the morphological changes in leukocyte in the form of band shaped and segmented neutrophils were also observed in all the age groups of Highlander camels (Table 3; Fig. 1).

**Table 3:** Mean (±SEM) and reference ranges of haematological parameters in Bactrian camels at high altitude

S. No.	Haematological parameters	Young age (2–4 years)	Adult age (5–10 years)	Old age (11–20 years)	Reference range at low altitude^a^	Reference range at high altitude^b^
1.	Hemoglobin (g/dl)	15.26 ± 0.92	14.61 ± 0.64	14.56 ± 0.51	11.1–17.4	12.8–17.5
2.	ESR value (mm/hr)	0.91 ± 0.03	0.91 ± 0.04	0.76 ± 0.0.07	0.0–1.0	0.63–1.03
3.	Packed cell volume (%)	41.0 ± 2.0	39.83 ± 0.54	40.0 ± 1.14	25.0–39.0	37.0–44.0
4.	Erythrocytes (✕10^6^/ mm^3^)	11.61 ± 0.14	9.90 ± 0.25	10.29 ± 0.61	8.5–13.4	9.11–12.13
5.	Leukocytes (✕10^3^/mm^3^)	16.16 ± 1.3	16.72 ± 0.05	15.52 ± 0.07	8.6–16.5	13.25–18.75
6.	Platelets (✕10^5^/ul)	8.23 ± 0.20	7.75 ± 0.16	8.12 ± 0.45	2.2–5.26	6.7–9.2
7.	Neutrophils (%)	53.3 ± 2.96	56.5 ± 2.09	56.6 ± 0.92	55.0–79.0	49.0–64.0
8.	Banded Neutrophils (%)	25.45 ± 1.08	23.24 ± 0.37	23.9 ± 1.02	–	–
9.	Lymphocytes (%)	38.0 ± 1.52	36.3 ± 2.27	35.8 ± 1.11	18.0–33.0	28.0–44.0
10.	Monocytes (%)	4.6 ± 1.20	3.83 ± 0.31	4.2 ± 0.58	0.0–4.0	3.0–7.0
11.	Eosinophils (%)	3.33 ± 0.33	2.5 ± 0.22	2.8 ± 0.37	0.0–9.0	2.0–4.0
12.	Basophils (%)	1.0 ± 0.0	1.0 ± 0.0	1.0 ± 0.0	0.0–1.00	1.0–2.0

^a^Reference range taken from Elitok and Cirak, (2018); ^b^ Reference range calculated based on present study; SEM- Standard Error Mean

The biochemical findings revealed a significant decrease in serum total protein (TP) and increase in triglycerides (TG) in a young age group as compared to an adult and old age groups (*p* < 0.05; Table 4). However, no significant variation was detected in ALT, albumin, AST, glucose, urea, creatinine, calcium, magnesium, and iron levels among the different age groups (*p* < 0.05; Table 4). Although, the ALT and blood urea level were within the normal ranges as reported in the literature. Similarly, the total protein, AST, albumin, creatinine, calcium, magnesium, and iron levels were within the range, but their values changed towards the higher side of normal range. Interestingly, total serum glucose levels were within the range of 45.00 to 70.00 mg/dl, which is very low as compared to a single hump camel (*C. dromedarius*), however within the range of 100 to 150 mg/dl [9].

**Table 4:** Mean (±SEM) and reference ranges of biochemical and antioxidant parameters in Bactrian camels at high altitude

S. No.	Biochemical parameters	Young age (2–4 years)	Adult age (5–10 years)	Old age (11–20 years)	Reference range at low altitude^**‡**^	Reference range at high altitude^**#**^
1.	Albumin (g/dl)	3.3 ± 0.05	3.58 ± 0.08	3.54 ± 0.09	2.8–3.3	3.2–3.9
2.	Calcium (mg/dl)	9.56 ± 0.22	9.61 ± 0.13	9.53 ± 0.11	6.4–10.3	9.12–10.05
3.	Creatinine (mg/dl)	2.3 ± 0.1	3.0 ± 0.25	3.1 ± 0.18	0.85–2.5	2.2–3.9
4.	Alanine aminotransferase (U/L)	14.10 ± 2.65	13.01 ± 1.62	11.6 ± 0.94	8.1–19.5	8.5–20.6
5.	Aspartate aminotransferase (U/L)	130.13 ± 15.4	104.8 ± 4.7	134.54 ± 19.2	69.0–98.0	91.5–208.70
6.	Triglycerides (mg/dl)	35.63 ± 4.14^b^	24.77 ± 1.29^a^	24.47 ± 1.58^a^	NR	18.38–43.65
7.	Iron (mg/L)	1.46 ± 0.12	1.25 ± 0.12	1.38 ± 0.17	0.83–1.5	0.8–1.8
8.	Magnesium (mg/dl)	2.36 ± 0.08	2.32 ± 0.16	2.33 ± 0.15	1.8–2.3	1.84–2.72
9.	Total Protein (g/dl)	5.91 ± 0.09^a^	6.77 ± 0.14^b^	6.81 ± 0.23^b^	5.5–7.0	5.73–7.62
10.	Urea (mg/dl)	46.87 ± 6.07	45.4 ± 3.19	51 ± 5.22	15.5–61.26	34.13–66.7
11.	Glucose (mg/dl)	67.36 ± 0.49	58.78 ± 3.34	62.62 ± 4.1	NR	45.73–77.8
	**Antioxidant parameters**					
1.	DPPH scavenging activity (%)	29.39 ± 5.37	38.78 ± 2.81	33.45 ± 2.95	NR	18.8–49.6
2.	FRAP assay (μmol/L)	175.0 ± 16.21	214.5 ± 25.84	209.14 ± 32.61	NR	148.7–337.6
3.	Uric acid (mg/dl)	0.13 ± 0.03	0.11 ± 0.01	0.16 ± 0.04	0.3–0.7	0.1–0.3

‡Reference range taken from Elitok and Cirak, (2018); # Reference range calculated based on present study; NR - Not reported; SEM- Standard Error Mean; ^a,b^ - Mean values bearing the same superscripts in a column do not differ significantly (*p* > 0.05)

Further, our result showed no significant variation in oxidative stress markers such as DPPH and FRAP among the different age groups (*p* < 0.05; Table 4). Though, their level was higher than the Lowlander camels’ viz. FRAP 214.15 ± 25.84 μmol/L and DPPH 38.78 ± 2.81%. Likewise, the uric acid concentrations revealed no significant variation among the different age groups (Table 4; *p* < 0.05), although, their levels were lower than the normal range of low altitude camels.

## Discussion

Our results show that the Highlander double hump camels are physiologically more active than the Lowlander double hump camels as shown by the increase in respiration rate, which results in hyperventilation, an important adaptive need under high altitude conditions. The variances in rectal temperature were towards the higher range as compared to Lowlander camels, despite extreme cold conditions, this might be due to high metabolism in double hump camel. Further, this high body temperature could be due to adaptive changes in energy metabolism and hepatic activities under cold and hypobaric hypoxia conditions. Similar observations have also been recorded in earlier reports on camels by Higgins & Kock [13], Tefera [14], and Al-Haidary et al. [15]. Higgins and Kock [13] studied the normal clinical parameters in the camel under field conditions and stated that a normal and healthy camel generally has a body temperature between 36 to 37 °C during daytime. Therefore, our result on physiological responses of the double hump camel indicates an adaptive change to the environmental condition of high altitude (Table 1). Hence, this new reference range of physiological parameters for Highlander’s double hump camel can be used for health monitoring.

In addition, the present findings revealed variation in different morphometric parameters except skin thickness and face length among the age groups, which could be due to age based different in growth pattern. So far there are no reports on the evaluation of morphometric parameters and their reference ranges in Bactrian camels. Although, few studies have analysed morphological diversity in Dromedarian camels to identify homogeneous groups depending upon their confirmation [13, 16, 17]. So, the present study is the first ever report on the estimation of reference intervals for morphometric parameters in different age groups of Bactrian camels of high altitude. These morphometric parameters and their reference ranges can be helpful in the overall evaluation of the animal’s health and selection of elite breeds for logistics purpose. The skin thickness and hump size are other important criteria during adaptation, because these parameters depend on the subcutaneous deposition of adipose tissue, which are the primary source of non-glucose energy substrates in animals adapted to a cold environment prevalent at high altitude.

Similarly, the present study on erythrocyte morphology indicated oval shaped and non-nucleated erythrocyte in high altitude camels as shown in Fig. 1, which help camel in resisting osmotic variation without damaging cell-membrane under cellular water stress and carry more oxygen [18]. Further, Hb concentrations in camels were slightly higher than the other domestic animals because of the high concentration of the erythrocyte. Moreover, increase in erythrocyte count, Hb, and PCV values indicate an adaptive change in oxygen transport under hypobaric-hypoxia for carrying out normal metabolic functions. The erythrocytes are enormously expansible and can expand to twice of their initial volume upon hydration, which were clear in the present study [19]. Likewise, the increase in leukocyte count could be due to stress factors prevalent at high altitude hypoxia conditions, which triggers an immune response for homoeostasis maintenance [18]. Further, we also observed higher morphological changes in neutrophils in the form of a band and segmented shape cells, these imply immunosuppression under high altitude stress. Therefore, more neutrophils are required to counter stress-induced ailments and microbial infection [13].

Since, these Highlander Bactrian camels are bred and reared for several generations under high altitude stress conditions, all these changes are likely have acquired by camels in response to high altitude climatic conditions over several generations. The high altitude cold stress could be responsible for the increased production of erythropoietin hormone, which further causes an increase in erythrocyte count at high altitude [20]. Similarly, El-Nouty et al. [21] also revealed that hypobaric-hypoxia condition at high altitude triggers the release of erythrocyte-stimulating factor, which increases erythrocyte count. Hence, increase in erythrocyte count in the present study is directly responsible for the increase in PCV and Hb levels in blood. Further, an increase in platelet count is associated with the stimulation of platelet precursor cells in response to hypoxic conditions at high altitude. The haematological parameters are an important indicator of health status in animals [22], hence changes in some parameters play the crucial role in assessing the camel health and performance in response to the environment. Zongping et al. [1] have studied the haematological parameters in Bactrian camels in China, at an altitude of 1200 m above msl, for clinical interpretation of data and revealed significant variation. Consequently, Elitok and Cirak, [9] have compared the reference values of haematological and blood biochemical parameters in Bactrian and Dromedary camels from the low altitude region. However, detailed studies of haematological parameters and their reference ranges have not been carried out so far in Highlander camel.

In addition, AST is a commonly used marker for hepatocyte injury. The high serum AST level in high altitude groups indicates that the liver is under continuous stress at a high altitude region, which releases more AST level in blood to adapt to the prevailing environment [23]. Since, liver metabolism is a vital physiological process to maintain body homoeostasis and necessary cellular metabolism under stressful conditions. Likewise, the creatinine level in the present study is towards higher range, which shows more active renal metabolism, another adaptive mechanism of camel for water conservation to prevent dehydration under high altitude conditions [24].

Further, our results revealed a very interesting observation on blood sugar levels, which was significantly lower. These findings advocate that high altitude seems to be the main reason for the increase in ATP fuel dependency from non-glucose energy substrate in Highlander camel. Under cold stress, non-glucose energy substrates are more important and preferred for ATP generation [25, 26]. Hence, at high altitude, the glucose based energy diets are avoided and protein-fat based diets are preferred as an adaptation mechanism for cellular energy generation. Earlier studies reported that exposure to high altitude region initially leads to a transient increase in glycaemic index, a possible adaptive mechanism to low energy level in Highlander camel [25–27]. However, a prolonged and continuous exposure to high altitude lowers glycaemia [25]. Furthermore, high altitude climate may be causing modulation of biochemical parameters, which is an important indicator of metabolic adaptation behaviour of Highlander camel [7, 9, 28]. Another factor could be the diversity in pasture flora, as high altitude plants are rich in secondary metabolites because of prevailing extreme climatic conditions. Since, camels graze on a hilly valley and plains which are flourishing with various herbal flora and especially seabuckthorne plant leaves. These plants are rich in many flavonoids and alkaloids phytomolecules having antioxidant properties [29]. Therefore, these phytomolecules may have beneficial effects on body metabolism and health of native livestock [29], including double humped camel through modulating body antioxidant status. However, these factors need detailed investigation on how different phytomolecules affect the native camel’s health and productivity. The Bactrian camels are one of the hardest animals of high altitude Leh-Ladakh, they can work comfortably and carry loads in this extreme climatic conditions. Hence, present study brought one of the important findings on Highlander double humped camel that their low blood glucose level is an adaptive biochemical mechanism for the high altitude condition.

Additionally, our results show high serum antioxidant levels (DPPH and FRAP) in all high altitude groups, which may have helped in reducing their cellular damage induced by oxidative stress under high-altitude stress conditions [30]. Hence, this better antioxidant level might have protected important biomolecules like DNA, RNA, proteins, carbohydrates and lipids from oxidative damage. These results suggest the importance of evaluation of antioxidant parameters for health monitoring and endurance studies. Thus DPPH assay is used to analyse the percentage of radical scavenging activity and antioxidant potential of serum by donating hydrogen to free radicals [31]. Whereas, a FRAP evaluates the total antioxidant capacity of serum by reducing ferric to ferrous ions [32]. Furthermore, monitoring of blood uric acid is also necessary for status of oxidative stress on animals. Interestingly, uric acid shows both antioxidant and free radical scavenging activities in domestic animals [33]. Thus, the lower value of uric acid as compared with Lowlander camel could be due to low cellular purine metabolism and normal renal function in Highlander double humped camel.

Nevertheless, the current study helped in understanding the morphological, physiological, haematological, and biochemical profiling of Highlander camels. There is no literature available on estimation of reference values of haematology and serum biochemistry of high altitude Bactrian camel. So, this study provides the reference ranges of these parameters, which will serve as a baseline for interpretation of the physiological changes in camel at high altitude. The reference range intervals in the present study showed the wide variations for different parameters. These variations could be due to diversity in animal breed, environment factors, and altitude effect [10, 34]. However, all these parameters essentially need to be within the normal range in healthy camels for deployment of the Army for patrolling and transportation at high altitude.

The main limitation of the present study is a small sample size of camels, which was because of the limited availability of high altitude camels in Nubra Valley, Leh-Ladakh. As, total population is only 219 nos camels, which are distributed in 59,146 km^2^ high hilly mountains, open barren areas of Ladakh, and so only a few camels were available at the farmer’s house. Because of these issues, control groups were also not available in this study for better comparison with high altitude Bactrian camels. However, we have used control reference ranges from the already available literature report of Elitok and Cirak [9] and these are accepted ranges. Thus, this study will be a reference for all the future research on the double humped camel of the high altitude region and further may be helpful in improving their husbandry practices and health management to increase their population in the high altitude regions.

## Conclusion

This study brought new knowledge on morphometric measurements, physiological, haematological, and biochemical parameters of Highlander double humped camel. In addition to this, the present finding revealed the deviation in Physio-biochemical and haematological parameters from the Lowlander double humped camel, an essential indicative of adaptation to high altitude conditions. Furthermore, this study concluded that the Bactrian camel has an effective, low-glucose energy metabolism and hypoglycaemic level as an adaptive mechanism for high altitude stress. Hence, these findings may be used for establishment of reference ranges for Highlander Bactrian camels, which will serve as a baseline for further studies using large sample sizes for interpretation of all physiological changes in camel.

## Methods

### Animals and sampling procedure

The present study was carried out at DRDO-Defence Institute of High Altitude Research (DIHAR), Ladakh at 3291 m mean sea level. All animal experiments were performed as per the regulations of the animal ethics committee of the DIHAR, C/o 56 APO, Leh-Ladakh. Total fourteen (*N* = 14) high altitude Bactrian camels (*Camelus bactrianus*) of different age groups (3 young age camels of 2–4 years, having 2 males and 1 female; 6 adult age camels of 5–10 years, having 5 males and 1 female; 5 old age camels of 11–20 years, having all males) were selected and divided into three age groups for sampling during winters. This study has a small sample size, which is the limiting factor in the reference range calculation as compared to data taken from a large sample size. This small sample size is because of the sparse distribution of approx 219 nos. Double humped camels in 59,146 km^2^ high hilly mountain open barren range of Ladakh, hence only a few camels were available at the farmer’s house for the study.

All the experimental camels were taken from Pack Animal Section, Animal Science Division, DRDO-Defence Institute of High Altitude Research, Leh-Ladakh, India and Farmers house of Nubra Valley, Leh-Ladakh. These camels were studied for morphometric measurements, recording physiological responses, and blood sampling at a farmer’s house and pack animal section of the Institute. Total 6–8 ml of blood samples were withdrawn from the jugular vein for separation of serum and analysis of different haematological and biochemical parameters. Thereafter, all the experimental results were compared with Lowlander Bactrian camels as reported by Elitok and Cirak [9]. After the experiments, all the camels were kept on the farmer’s farm and pack animal section of the Institute for further rearing and breeding.

All the studied camels were clinically healthy and in good body condition during the sampling period. The sampling of all the camels was performed in the morning when they were off feeding, further no load or riding were deployed during or prior 4–5 weeks of this study. This study has excluded pregnant and diseased or unhealthy animals. However, mostly male camels were used in the present study. All these camels were on similar feeding systems like pasture grazing, stall feeding, and left-out household grains or concentrate feeding. These exclusions, condition has been considered before the design of this study to avoid any ambiguity on physiological, biochemical, and haematological observations.

### Determination of morphometric and physiological parameters

All the body morphometric measurements of camel were recorded using measuring tape (Freemans 5 m × 16 mm) as described by Yosef et al. [16]. The thickness of the shank and abdomen skin was measured using Vernier Caliper (MGW Precision VCF200). The physiological parameters such as heart rate (beats/min) and respiration rate (beats/min) were recorded by Stethoscope and by manual counting through nasal orifice, respectively. The skin and rectal temperature was recorded by Laser Digital Infrared Thermometer (Phoenix, ST350).

### Determination of haematological parameters

Haemoglobin (Hb) concentration was estimated by Sahli’s method [35], whereas packed cell volume was analysed using Haematocrit capillary tube according to Schalm et al. [36]. Whereas, total erythrocyte and leukocyte count was performed by Haemocytometer under phase contrast microscope [36]. The differential leukocyte count (DLC) was estimated as per Giemsa stain method under light microscope [36]. Further, erythrocyte sedimentation rate (ESR) and platelet count was determined by Wintrobe tube Method [37] and sodium citrate dilution method [38], respectively.

### Determination of biochemical and antioxidant parameters

Serum glucose, total protein (TP), albumin, calcium, creatinine, alanine aminotransferase (ALT), aspartate aminotransferase (AST), uric acid, urea, triglycerides, iron, and magnesium were estimated using commercially available kits with automated serum biochemical analyser (BS-120, Mindray Medical International Ltd.). Ferric Reducing Antioxidant Power (FRAP) assay was used to analyse the serum total antioxidant status of the sample [32], whereas free radical scavenging activity was determined by 2,2-diphenyl-1-picryl-hydrazyl-hydrate (DPPH) radical scavenging method [31, 39].

### Statistical analysis

To establish reference intervals, all the morphological, physiological, haematological, and biochemical variables were included as continuous variables, and their distributions were determined. Descriptive statistics were performed with upper and lower 95% confidence intervals for all the variables. The distributions of all the variables were assessed using histograms and QQ-plots, as if they had a Normal distribution. Those with an approximate normal distribution were kept on the direct scale, whereas Box-Cox transformation method was used for variables that had non-normal distribution.

Further, comparison between the age groups were performed on normally distributed variable and transformed variables using One Way Analysis Of Variance (ANOVA). A Tukey’s Honest Significance Difference (HSD) test was used to analyse the data for significant difference between the age groups. The statistical analysis was performed using SPSS software (Version 24, IBM Corporation, USA). All the results have been represented as mean and ± standard error mean (SEM) and < 0.05 *P* values were considered as significant.

## Data Availability

Data generated or analysed during this study are included in this paper and can be made available by the corresponding author upon substantiated request.

## Abbreviations

ALT: Alanine aminotransferase
AST: Aspartate aminotransferase
bpm: beats per minute
DLC: Differential leukocyte count
DPPH: 2,2-diphenyl-1-picryl-hydrazyl-hydrate
ESR: Erythrocyte sedimentation rate
FRAP: Ferric reducing antioxidant power
Hb: Hemoglobin
PCV: Packed cell volume
SEM: Standard error mean
TP: Total protein
TG: Triglycerides
